# Cytokine and Chemokine Responses of Peripheral Blood Mononuclear Cells from Dogs Infected with *Mycobacterium bovis*

**DOI:** 10.3390/pathogens14010017

**Published:** 2024-12-30

**Authors:** Tyler Morrison, Danielle A. Gunn-Moore, Jayne C. Hope, Conor O’Halloran

**Affiliations:** Roslin Institute and Royal (Dick) School of Veterinary Studies, University of Edinburgh, Midlothian EH25 9RG, UK; tyler.morrison@ed.ac.uk (T.M.); danielle.gunn-moore@ed.ac.uk (D.A.G.-M.); jayne.hope@roslin.ed.ac.uk (J.C.H.)

**Keywords:** *Mycobacterium bovis*, canine, cytokine, chemokine

## Abstract

Mycobacterial infections are an important emerging zoonosis in companion animals for which diagnostic options remain imperfect, and the canine immunological response to these infections has been poorly investigated. We sought to further define the cellular response of peripheral blood mononuclear cells (PBMCs) from dogs infected with *Mycobacterium bovis*, as determined using a commercial interferon-gamma response assay (IGRA). To this end, PBMCs from healthy or infected dogs were collected. Serum samples were tested to further classify dogs as seropositive or seronegative for circulating antibodies against *M. bovis* using the DPP^®^ VetTB Assay, Idexx *M. bovis* antibody ELISA, and a novel purified protein derivative ELISA. Isolated PBMCs were stimulated with mycobacterial proteins (PPDB or ESAT-6/CFP-10), and 13 cytokines/chemokines were measured in the supernatant. These concentrations were determined using the CYTOMAG-90K MILLIPLEX MAP Canine Cytokine/Chemokine system. PBMCs from infected dogs released IFN-γ in response to stimulation, but this response was reduced in those that had seroconverted. Similarly, cells stimulated with PPDB secreted increased amounts of TNF-α when dogs were seronegative, but cells taken from seropositive dogs did not. Finally, the IL-18 response of seropositive dogs was reduced compared to those that were seronegative in response to PPDB, potentially suggesting that these dogs have a reduced macrophage functionality. This work demonstrates that the inflammatory cytokine response may wane following seroconversion with deleterious consequences for the host response. Overall, combining IFN-γ and TNF-α assessment during diagnosis may increase IGRA sensitivity, whilst further work is needed to better understand the prognostic and diagnostic implications of seroconversion in dogs.

## 1. Introduction

Human tuberculosis, caused by *Mycobacterium tuberculosis*, is amongst the leading cause for morbidity and mortality for any single infectious disease worldwide [1,2,3]. *M. tuberculosis* is one of a group of very closely related species within the *Mycobacterium tuberculosis* complex (MTBC), which is capable of causing tuberculosis (TB) in a wide range of species. The MTBC shares identical 16s RNA and has 99.5% sequence homology across the remaining genome. Those regions that discriminate them, so-called regions of difference (RDs), are deletions from parts of the genome that can encode a variety of genes including virulence factors; for example, the natural RD-1 deletion makes *M. microti* naturally less virulent than *M. bovis* [4].

Notably, MTBCs are important zoonoses and exist within a range of mammalian reservoir species. Specifically, *M. bovis* appears to display the greatest host promiscuity, being able to cause cases of zoonotic TB and being responsible for an estimated 140,000 cases of zoonotic TB each year, which resulted in approximately 11,400 deaths in 2020 [5]. *M. bovis* retains significant interest in the UK and other countries as the causative agent of bovine TB, where it inflicts a significant socioeconomic burden, as well as zoonotic risk. Furthermore, *M. bovis* sporadically infects companion animals, including cats, most frequently following ingestion or inoculation by hunting infected prey [6,7]; at present, it has been diagnosed in approximately 1% of UK feline pathology submissions [8]. Although rare, such infections present a known zoonotic risk [9].

TB in dogs has received less attention, although it is a re-emerging concern both as a sentinel for human *M. tuberculosis* infection as well as a source of exposure for other zoonotic MTBC members, e.g., *M. bovis* [10,11,12]. *M. bovis* retains significant potential for canine infection in endemic regions, with a large outbreak reported in a kennel of foxhounds in the UK [13]. Recognizing such infections can be challenging as dogs present with non-specific clinical signs, or signs considered to represent more frequently encountered illnesses [14]. Moreover, even when suspected, diagnosis can be challenging and relies on a constellation of findings including culture, nucleic acid amplification, and pathological findings. Reliable and timely non-invasive tests are therefore essential. 

The interferon-gamma (IFN-γ)-response assay (IGRA) has been developed as a non-invasive test for TB in a range of species including cattle, humans, cats, and dogs [13,15,16]. Following infection with MTBCs, host antigen-specific T-cells respond to mycobacterial antigens in vitro to produce IFN-γ, which can be detected to support a diagnosis [17].

An IGRA test was developed for the investigation and management of a large outbreak of *M. bovis* TB in an outbreak among working foxhounds [13]. This was used as the key test to diagnose individual hounds as it tests a cell-mediated response that is the predominant immunological response to any mycobacterial infection [18]. However, the cell-mediated response, sometimes referred to as anergy or “immune exhaustion”, can decline and correlates with the production of antibody, the generation of Th2 cells, and progressive infection [19]. In the affected group of kenneled dogs, a contemporaneous serological test, the Dual Path Platform (DPP) VetTB test for Cervids (Chembio Diagnostic Systems Inc., Medford, NY, USA) was used to identify antibody (seropositive) animals, as described in O’Halloran et al. [13].

The utility of the IGRA test has been expanded to distinguish some of the different mycobacterial infections in cats [20], and more recently to differentiate active and latent TB in people where the IGRA has greater sensitivity and specificity compared to previous test methods [21,22,23]. The ability to distinguish different TB states has emerged by quantifying additional cytokines, such as interleukin (IL)-2, alongside IFN-γ within the supernatant from IGRA cells [22], and there is growing interest in defining specific cytokine and chemokine responses to mycobacterial antigens. In this regard, circulating cytokines in serum/plasma have previously been shown to distinguish cats with mycobacterial infections from cats hospitalized for other reasons, whilst tumor-necrosis factor (TNF)-α and platelet-derived growth factor (PDGF)-BB were able to distinguish cats infected with *M. bovis* from *M. microti* [24]. There is a paucity of evidence for cytokine and chemokine responses to MTBC infections in dogs.

We therefore aimed to evaluate the antigen-specific cytokine and chemokine responses of canine peripheral blood mononuclear cells (PBMCs) from dogs with and without *M. bovis* infection.

We hypothesized that PBMCs from infected dogs would have a distinct cytokine and chemokine profile that would shed light on the canine immune response to *M. bovis* and may guide modifications or additions to the IGRA to improve the sensitivity of *M. bovis* diagnosis in dogs.

## 2. Materials and Methods

### 2.1. Animals

Blood samples were obtained from client-owned, kenneled foxhound dogs that were tested to facilitate the control of an outbreak of TB caused by *M. bovis* infection [13]. Heparinized whole blood (5 mL) was collected from each dog and transported at ambient temperature for analysis within 18 hours. Dogs were tested using the IGRA, as previously reported [13], and were considered *M. bovis*-infected if they met the test-positive criteria with respect to the control samples (IGRA+) [13]. Dogs with an initial negative IGRA test were confirmed as TB-negative with a second test performed 60 days later. 

Dogs concurrently underwent serological testing for *M. bovis* using three serological assays. The DDP test, as described by O’Halloran et al. [12], at the time of the outbreak and then subsequently with two additional serological assays applied to dogs for the first time—the Idexx *M. bovis* antibody ELISA (Idexx Laboratories, Inc., Westbrook, ME, USA) performed with minor modifications to detect canine antibodies, and an in-house comparative PPD ELISA (O’Halloran et al. 2024 manuscript under peer-review, available via Preprint.org ID 141207).

Dogs testing negative to all three serological tests in addition to the IGRA were considered uninfected (IGRA−; uninfected controls). IGRA + dogs were subdivided according to their serological status such that IGRA + seronegative dogs were still considered TB infected but labeled IGRA+/seronegative. If dogs were positive to one or more serological test, they were considered and assigned as IGRA+/seropositive.

This study was conducted following approval from the R(D)SVS Veterinary Ethical Review Committee at the University of Edinburgh, and all relevant guidelines and regulations were adhered to throughout.

### 2.2. Cell Stimulation

The PBMCs were isolated using a Histopaque 1077 cell separation gradient (Sigma-Aldrich, Gillingham, UK), as previously described, for performing the canine IGRA test [13]. Isolated cells were then stimulated either with purified protein derived from *M. bovis* (PPDB) at a final concentration of 10 µg/mL (Lelystad Prionics, Lelystad, The Netherlands) or a cocktail of early secreted antigenic target-6KDa (ESAT-6) and culture filtrate protein-10KDa (CFP-10) (ESAT-6/CFP-10) at a final concentration of 5 µg/mL (Lionex Diagnostics and Therapeutics, Braunschweig, Germany) in complete culture media (RMPI 1640 media containing 100 µg/mL L-glutamine, 10% fetal bovine serum, 100 µg/mL penicillin, 100 U/mL streptomycin, 5 × 10^−5^ M mercaptoethanol, and non-essential amino acids). An unstimulated control (complete culture media only) was used for comparison. Cells were incubated for 4 days at 37 °C/5% CO_2_ and antigen-stimulated PBMC supernatants were collected and retained at −80 °C prior to analysis.

### 2.3. Cytokine and Chemokine Measurements

Cytokine and chemokine concentrations were measured in supernatant samples diluted at 1:2 in RPMI 1640 cell culture media (Gibco, Thermo Fisher Scientific, Paisley, UK) using a commercial, canine-specific, antibody-coated microsphere-based multiplex cytokine immunoassay that was shown to be able to quantify 13 cytokines contemporaneously using 25 μL of each sample (CYTOMAG-90K MILLIPLEX MAP Canine Cytokine/Chemokine Magnetic Bead Panel, Premix 19 Plex kit, MERCK Millipore Corporation, Billerica, MA, USA).

The following cytokines were measured: granulocyte-macrophage colony-stimulating factor (GM-CSF), IFN-γ, interleukin (IL)-2, IL-6, IL-7, IL-8, IL-10, IL-15, interferon gamma-induced protein (IP)-10, IL-18, keratinocyte-derived chemokine-like protein (KC-like), monocyte chemoattractant protein 1 (MCP-1, also known as CCL2), and tumor necrosis factor (TNF)-α. All samples, standards, and quality controls were assayed in accordance with the manufacturer’s instructions and in duplicate, the mean of which was used to calculate the difference between antigen-stimulated and unstimulated control samples. Plates were read on a multiplex plate reader (Luminex™ 200™ Instrument System, Thermo Fisher Scientific^®^, Waltham, MA, USA).

### 2.4. Statistical Analysis

Cytokine and chemokine concentrations were calculated using the plate reader companion software (xPONENT^®^ basic plus version 4.2.1324.0, Thermo Fisher Connect Platform, Waltham, MA, USA) and expressed as ρg/mL. Values that fell below the limit of detection were assigned a concentration of 0 ρg/mL for the purposes of analysis. Following acquisition, the change in cytokine/chemokine concentration in the supernatant following stimulation was calculated by subtracting the concentration of the unstimulated control from the stimulated cells of the same animal. Data were normalized arbitrarily to the mean of the uninfected controls for each cytokine/chemokine. The normality of the data was assessed using the D’Agostino and Pearson omnibus test. Most data were not normally distributed and due to the negative values, were assessed using non-parametric assessments rather than being transformed. To this end, the effect of infection status was assessed using the Kruskal–Wallis test, with Dunn’s multiple comparisons used to assess the individual groups. Statistical analysis was performed using GraphPad^®^ Prism^®^ v. 10.0 for MacOS (GraphPad Software, Boston, MA, USA).

## 3. Results

### 3.1. Patient Characteristics

Blood was obtained from 60 of the 164 foxhounds housed in a single premises that experienced a fulminant outbreak of *M. bovis* TB (as described previously, [13]). Twenty-two (37%) were IGRA-negative (on two occasions, 60 days apart) and seronegative meaning they were classed as uninfected with *M. bovis* (control group). Meanwhile, 38 dogs (63%) were IGRA + and considered infected; in this IGRA + group, 16 (42%) were IGRA +/seropositive and 22 (58%) were IGRA+/seronegative.

### 3.2. Cytokine and Chemokine Concentrations

#### 3.2.1. PPDB-Stimulated Cells

**Figure 1:**
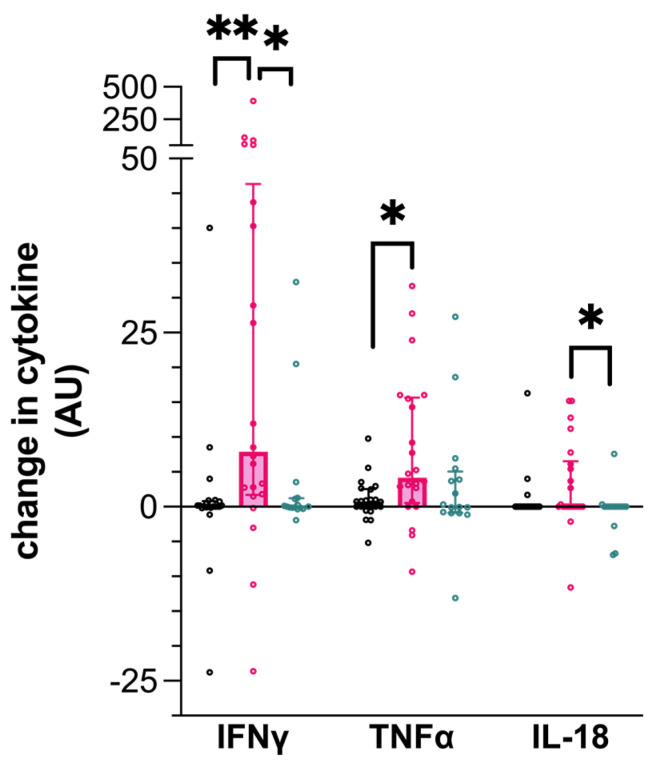
Cytokine production by canine peripheral blood mononuclear cells (PBMCs) following stimulation by purified protein derived from *Mycobacterium bovis* (PPDB). Data represent the individual values of the antigen-specific cytokine concentration for each dog, along with the median ± interquartile range of the group. Dogs are grouped as IGRA+/seronegative (pink), IGRA+/seropositive (turquoise), or as uninfected control (black). ** *p* < 0.01; * *p* < 0.05; AU—arbitrary units.

In keeping with IGRA + status, IFN-γ production was significantly greater from PBMC isolated from the IGRA+/seronegative dogs following PPDB stimulation compared to uninfected control dogs (0.025, CI −0.06–0.76 ρg/mL; 7.9, CI 1.8–43.74 ρg/mL; *p* = 0.005, Figure 1). This increase was not seen when comparing PPDB-stimulated PBMCs from IGRA+/seropositive dogs to the control dogs (0.02, CI −0.16–1.29 ρg/mL; *p* = 0.0156). 

Similarly, TNF-α production was significantly greater following the PPDB stimulation of PBMCs isolated from IGRA+/seronegative dogs (0.74, CI −0.04–2.51 ρg/mL; 4.19, CI 0.74–15.53 ρg/mL; *p* = 0.033, Figure 1), compared to the uninfected dogs. In comparison, IGRA+/seropositive dog PBMCs expressed intermediate TNF-α concentrations following stimulation with PPDB, but this was not statistically different from the control dog PBMCs.

IL-18 production by PPDB-stimulated PBMCs was significantly lower in seropositive infected dogs (IGRA+/seropositive) compared to dogs that were IGRA+/seronegative (0.0, CI 0–0 ρg/mL; 0.0, CI 0.0–6.1 ρg/mL; *p* = 0.0407, Figure 1) but was not different from the uninfected control dogs.

IP-10 was undetectable in all canine PBMCs stimulated with PPDB. The concentration of the remaining cytokines tested in the assay was not significantly different between any of the three groups following the PPDB stimulation of PBMCs.

#### 3.2.2. ESAT-6/CFP-10-Stimulated Cells

**Figure 2:**
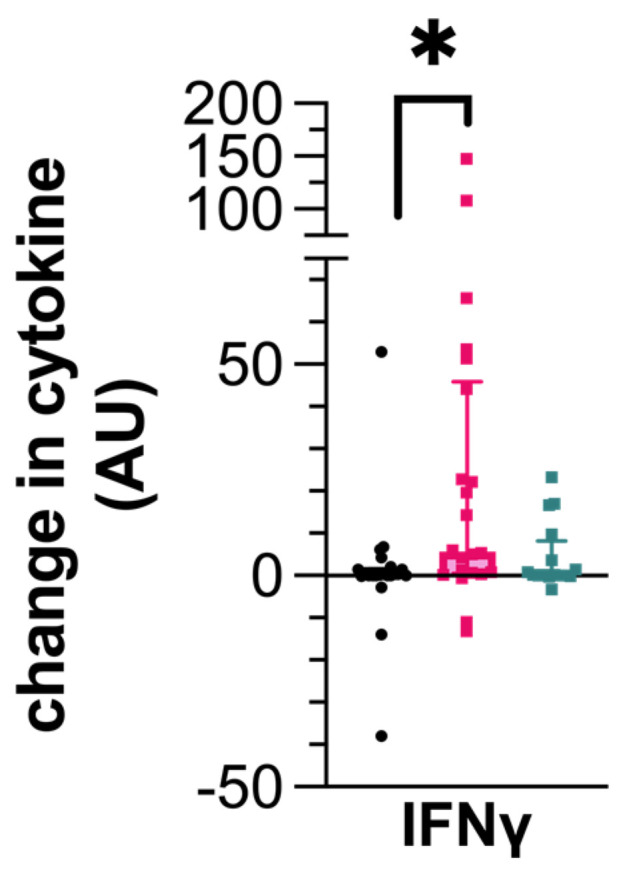
Cytokine production by canine peripheral blood mononuclear cells (PBMCs) following stimulation with a cocktail of ESAT-6/CFP-10. Data represent the individual values of the antigen-specific cytokine concentration for each dog along with the median ± interquartile range of the group. Dogs are grouped as IGRA+/seronegative (pink), IGRA+/seropositive (turquoise), or as uninfected control (black). * *p* < 0.05; AU—arbitrary units.

Following ESAT-6/CFP-10 stimulation, PBMCs from IGRA+/seronegative dogs produced more IFN-γ compared to the uninfected control dogs (0.255, CI 0–1.45 ρg/mL; 5.675, CI 0.59–44.12 ρg/mL; *p* = 0.0129, Figure 2). However, IFN-γ production from PBMCs of the IGRA+/seropositive dogs was not different to those dogs that were uninfected.

Like those PBMCs stimulated with PPDB, IP-10 was not detectable in any of the samples. The remaining cytokines were produced at similar concentrations across the dog groups with no significant differences.

## 4. Discussion

Canine TB following *M. bovis* infection can be occult with limited diagnostic options, yet poses a zoonotic risk to owners [10,14]. The production of IFN-γ by PBMCs or whole blood following exposure to mycobacterial antigens has been routinely used in the diagnosis of *M. bovis* in other species, and the assessment of other cytokines has more recently gained interest in defining an individual’s disease state or prognosis [22,25]. This study aimed to assess the change in a range of cytokines and chemokines in stimulated PBMCs from dogs diagnosed with canine TB.

In keeping with the IGRA results following conventional IFN-γ ELISA, IGRA + dogs demonstrated significant IFN-γ release in response to both mycobacterial antigen groups tested in this study. IFN-γ is a type II IFN cytokine secreted primarily by T-helper 1 (Th1) and natural killer (NK) cells in response to any mycobacterial infection. Among a range of mechanisms, IFN-γ activates the macrophage production of reactive nitrogen species to restrict mycobacterial growth [26]. A higher production of IFN-γ has been associated with latent TB infection (LTBI), where mycobacteria are contained within granulomata in a dormant and asymptomatic state [27]. Importantly, people with latent tuberculosis infection (LTBI) have a 5–10% lifetime risk of reactivation and active disease [28]. We demonstrated that dogs who were seropositive produced less IFN-γ than those that were seronegative.

The role for antibody-mediated protection against TB is controversial and whilst B-cell depletion studies demonstrated limited effect on disease outcomes in models of infection, more recent evidence has shown a role for the antibody enhancement of cell-mediated immunity against mycobacteria in a range of contexts [29,30]. Recently, it has become clear that the presence of specific combinations of serological targets in a patient can be helpful to distinguish against active and LTBI [31]. In this study, dogs needed to test positive via only one serological test to be considered seropositive, and future work should explore the potential range of antigens that are serological targets in the dog. However, the reduced IFN-γ released upon stimulation with PPDB in dogs that were seropositive raises interesting questions regarding their immunological response and associated relationship to infection outcome. Notably, seropositivity at the time of diagnosis of TB in human patients in South Africa was associated with a slow response to treatment, and suggests that specific seropositivity may also serve as a negative prognostic marker [32]. That seropositivity was associated with a blunted IFN-γ response in dogs infected with TB could allow for prognostication in the future and is an important observation particularly since choosing to treat pet dogs is a decision that carries an ethical dilemma. Whilst we cannot say whether seroconversion represents the progression of a disease state, or an intrinsic difference in the outcome in the dogs studied herein, there is clearly a need for a better understanding of the canine immunological response in such contexts.

Critically, the significant reduction in IFN-γ produced by seropositive animals could suggest that animals with a more advanced disease and who have moved from a more protective anti-mycobacterial Th-1-biased response to a more Th-2-mediated response, such as that which occurs during the progression of other mycobacterial diseases including *M. avium* subspecies paratuberculosis (MAP, also known as Johne’s disease) in cattle. This indicates a possibility that a potentially infectious animal may not be diagnosed using the reference standard test (IGRA) and lends even greater significance to the enhancement of diagnostics for what is already a challenging condition to identify.

In addition to IFN-γ, TNF-α is produced by macrophages and other cells to enact a range of host-protective functions and is required for protection against MTBC infections [33]. Alongside IFN-γ, TNF-α demonstrated a similar pattern of release from PBMCs from seronegative dogs infected with *M. bovis*, following stimulation with PPDB. TNF-α has been demonstrated to be essential in protecting mice from death following *M. tuberculosis* infection, where it is involved in generating granulomata [34,35]. Given that the pattern of TNF-α is like IFN-γ, it is likely that dogs have a similar capacity to produce T-cell-derived PPDB-stimulated IFN-γ and the subsequent release of TNF-α from monocytes. However, the same cannot be said following stimulation with the ESAT-6-CFP-10 cocktail, where there was no difference in TNF-α release following the stimulation of PBMCs derived from *M. bovis*-infected dogs. PPDB contains many peptides, all of which likely have the unique capacity to stimulate primed T-cells. This is in contrast to the more refined cocktail of ESAT-6/CFP-10, which comprises specific proteins released via the ESAT-6 (ESX)-1-type VII secretion system, which is not constitutively expressed [36]. Therefore, the likelihood of an antigen-specific response to ESAT-6/CFP-10 is reduced. Furthermore, in prior studies in cattle that were infected with *M. bovis*, individual animal responses to the peptide cocktails were not equivalent, with only around 20% of infected cattle mounting a response to ESAT-6/CFP-10 compared to PPDB [37].

IL-18 was heterogeneously released by PBMCs stimulated with PPDB from some dogs infected with *M. bovis*, and appeared to follow a pattern similar to that of IFN-γ and TNF-α, where induction was reduced in dogs that were seropositive. IL-18 is one of two key cytokines, alongside IL-12, that are thought to be important in the induction of Th1 cells and the release of IFN-γ [38]. Many cells can release IL-18, including monocytes, dendritic cells, and stromal cells, even after death [39]. Mice deficient in IL-18 have been shown to have enhanced susceptibility to *M. tuberculosis* infection via a Toll-like receptor (TLR)-2/TLR-4-dependent pathway. Without IL-18, the protective Th1 response was abrogated, leading to unrestricted mycobacterial growth and host death [40]. These data suggest a link between IL-18 and the antibody status of dogs with TB. Further work to better understand the role of IL-18, as well as the seroconversion and host outcomes in dogs is therefore required, particularly given its significance to monocyte–macrophage and dendritic cell function and activation states, which are deeply fundamental to the mycobacterial response.

Altogether, the results of this study suggest that the combination of the IFN-γ and TNF-α release from PBMCs derived from infected dogs may enhance the diagnostic capacity of commercially available IGRA testing. Furthermore, the distinct cytokine profile of dogs that have seroconverted raises important questions about the disease status and infection outcomes in such conditions that may be driven by differences in IL-18 production.

This study is strengthened by the use of a naturally infected population of dogs in an outbreak situation, allowing for a degree of variability control such as the fact that the hounds were the same breed, housed in a homogenous environment, and were of a restricted age range (range: 1–8 years; median: 4 years), which permits the dissection of the cytokine profile in this context. However, there are some limitations to our data. As stated, our data come from a single kennel group of foxhounds that are likely to be highly genetically related and therefore, the generalization of our data across canines more widely requires some caution. Furthermore, the samples were taken at a single time point and so whilst there are interesting cytokine responses following seroconversion, we are unable to determine whether this is due to a disease continuum; host or pathogen/dose factors and longitudinal infection challenge would be necessary to better understand this (though this would be ethically challenging).

Finally, the diagnosis of *M. bovis* and other mycobacterial infections are notoriously challenging in dogs. IGRA status has been previously shown to be a specific marker for infection in dogs [13] and has been validated in other species [15,16]. This study used IGRA-negative dogs as an uninfected control group although a full range of diagnostic tests including necropsy, mycobacterial culture, and PCR were not performed on these animals. We cannot therefore entirely exclude the possibility that a small number may have been exposed to, or infected with, *M. bovis*.

In conclusion, the results of this study demonstrate that the assessment of a combination of IFN-γ and TNF-α may enhance the sensitivity of the IGRA test in dogs to diagnose infection with *M. bovis*. Moreover, these data emphasize that the serological responses of dogs to *M. bovis* infection should be investigated further, given the potential to influence other diagnostic test responses that may lead to infected dogs generating negative results to the current standard-of-care test.

Additional studies into the immunological response of dogs to mycobacterial infections and *M. bovis* in particular require future research to enhance our currently limited diagnostic testing repertoire. This study suggests that a combination of antibody assessment along with IGRA tests that combine multiple cytokine responses (i.e., TNF-α, as well as the baseline IFN-γ) opens the door to further study into the prognostic and diagnostic power of different TB disease states in dogs.

## Data Availability

The original contributions presented in this study are included in the article. Further inquiries can be directed to the corresponding author.
